# Suppression of Ovarian Cancer Cell Growth by AT-MSC Microvesicles

**DOI:** 10.3390/ijms21239143

**Published:** 2020-11-30

**Authors:** Agnieszka Szyposzynska, Aleksandra Bielawska-Pohl, Agnieszka Krawczenko, Olga Doszyn, Maria Paprocka, Aleksandra Klimczak

**Affiliations:** Laboratory of Biology of Stem and Neoplastic Cells, Hirszfeld Institute of Immunology and Experimental Therapy, Polish Academy of Sciences, R. Weigla 12, 53-114 Wroclaw, Poland; agnieszka.szyposzynska@hirszfeld.pl (A.S.); aleksandra.bielawska-pohl@hirszfeld.pl (A.B.-P.); agnieszka.krawczenko@hirszfeld.pl (A.K.); olga.doszyn@hirszfeld.pl (O.D.); maria.paprocka@hirszfeld.pl (M.P.)

**Keywords:** ovarian cancer cells, ES-2, OAW-42, microvesicles, adipose tissue origin mesenchymal stem cells

## Abstract

Transport of bioactive cargo of microvesicles (MVs) into target cells can affect their fate and behavior and change their microenvironment. We assessed the effect of MVs derived from human immortalized mesenchymal stem cells of adipose tissue-origin (HATMSC2-MVs) on the biological activity of the ovarian cancer cell lines ES-2 (clear cell carcinoma) and OAW-42 (cystadenocarcinoma). The HATMSC2-MVs were characterized using dynamic light scattering (DLS), transmission electron microscopy, and flow cytometry. The anti-tumor properties of HATMSC2-MVs were assessed using MTT for metabolic activity and flow cytometry for cell survival, cell cycle progression, and phenotype. The secretion profile of ovarian cancer cells was evaluated with a protein antibody array. Both cell lines internalized HATMSC2-MVs, which was associated with a decreased metabolic activity of cancer cells. HATMSC2-MVs exerted a pro-apoptotic and/or necrotic effect on ES-2 and OAW-42 cells and increased the expression of anti-tumor factors in both cell lines compared to control. In conclusion, we confirmed an effective transfer of HATMSC2-MVs into ovarian cancer cells that resulted in the inhibition of cell proliferation via different pathways, apoptosis and/or necrosis, which, with high likelihood, is related to the presence of different anti-tumor factors secreted by the ES-2 and OAW-42 cells.

## 1. Introduction

Today, ovarian cancer is one of the most dangerous types of cancer in women. This is associated with a lack of screening tests and late diagnosis. Moreover, the disease has no symptoms in the early stages. Currently, various ovarian cancer therapies are used depending on the histological type of ovarian cancer, its stage, and the patient’s predisposition. Standard treatment is a surgery combined with platinum-based chemotherapy [1]. Clinical trials focus primarily on an anti-angiogenic strategy [Vascular endothelial growth factor (VEGF) inhibition] or on modulating the immune system [2]. An extremely important field in oncology is research focused on cancer stem cells (CSCs). Cancer stem cells constitute a small population of tumor cells and play an important role in metastasis. Moreover, these cells are resistant to widely used drugs, which often leads to tumor recurrence [3]. Thus, a search for effective factors is needed that inhibit the biological activity of CSCs.

Mesenchymal stem/stromal cells (MSCs) are multipotent cells that reside in the majority of human tissues and organs, and in steady-state conditions, are responsible for the maintenance of tissue homeostasis [4,5]. Cells with MSC characteristics can be isolated from various source tissues, such as bone marrow, adipose tissue, dental pulp, skin, skeletal muscle, or perinatal tissues, including the umbilical cord, cord blood, Warton’s jelly, and amniotic fluid. The tissue source of MSCs affects their cellular phenotype and biological properties [6]. MSCs and their derivates are a promising tool in clinical applications thanks to their high proliferative potential, longevity, and immunomodulatory properties [7,8].

Extracellular vesicles (EVs), such as exosomes and microvesicles (MVs), play an important role as mediators of cell-to-cell communication [9]. EVs are released by all normal, apoptotic, and neoplastic cells [10]. The transport of bioactive cargo, such as proteins, lipids, or nucleic acids, into the recipient cells may affect their phenotype and biological activity [11].

The tumor microenvironment consist not only of tumor cells, but also fibroblasts, smooth muscle cells, immune cells, endothelial cells, and mesenchymal stem cells [12]. Cell-to-cell communication in tumor niches takes place through direct contact between the surrounding cells and gap junctions or through the paracrine activity of the cells (e.g., the release of soluble factors or EVs).

The effect of EVs derived from MSCs of different tissue origin on cancer cells is not well understood. Different studies have confirmed the pro-tumorigenic [13] or anti-tumorigenic activity [14] of EVs derived from MSCs on ovarian cancer cells. This effect depends on the origin of the MSCs, methods of EV isolation, and tumor type [15].

The aim of this study was to examine the effect of MVs derived from immortalized human MSCs of adipose tissue origin (HATMSC2-MVs) on the biological activity of two ovarian cancer cell lines: ES-2, representing poorly differentiated ovarian clear cell carcinoma, and OAW-42, representing ovarian cystadenocarcinoma, with different genetic backgrounds and therapeutic responses. These two cell lines were characterized according to their phenotype and the secretion profile of cytokines and trophic factors released in response to MV treatment. Moreover, we investigated the proliferation and cell death processes/pathways (apoptosis and necrosis) of ovarian cancer cells in the presence of different ratios of HATMSC2-MVs and target cells.

## 2. Results

### 2.1. Characterization of HATMSC2-Derived MVs

**Figure 1:**
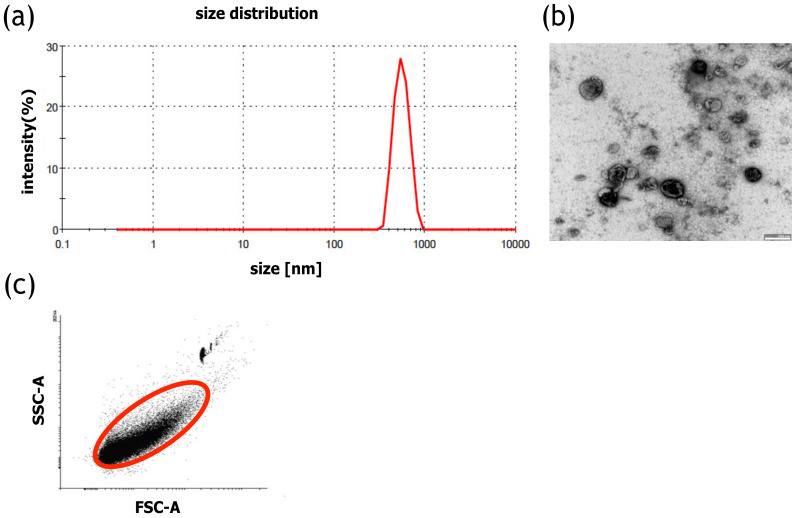
Characteristics of HATMSC2-MVs (**a**) Representative HATMSC2-MV size distribution histogram obtained using dynamic light scattering analysis. (**b**) Representative transmission electron microscopy image of HATMSC2-MVs, bars represent 500 nm. (**c**) Representative dot plot showing forward scatter (FSC) vs. side scatter (SSC). Gate R-1 shows the population of HATMSC2-MVs, and gate R-2 represents the counting beads. HATMSC2-MVs—microvesicles derived from immortalized human mesenchymal stem cells of adipose tissue origin.

Size distribution of MVs was analyzed using dynamic light scattering (DLS). In the histogram, a single distinct peak characteristic for MVs was observed, confirming the presence of a homogenous population of MVs. The average size of MVs, assessed using DLS, was 456 nm (Figure 1a). The size of individual MVs was confirmed using transmission electron microscopy (TEM) imaging (Figure 1b). 

Isolation efficiency, using the flow cytometry method, revealed that the average number of MVs was 172 × 10^6^ MVs/mL (Figure 1c). A Bradford assay was performed to estimate the protein concentration within the MVs, and the average protein concentration was assessed at 169.8 µg/mL.

### 2.2. Surface Marker Analysis of HATMSC2 Cells and HATMSC2-MVs

**Figure 2:**
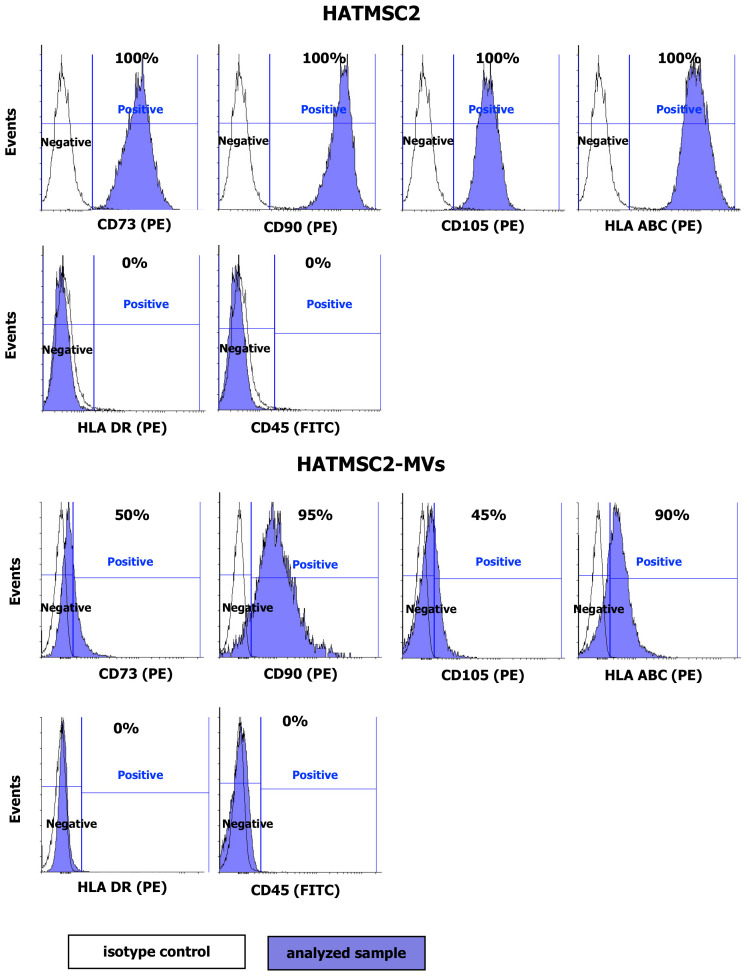
Representative flow cytometry histograms of flow cytometry analysis of mesenchymal stem cells markers (CD73, CD90, CD105, HLA ABC, HLA DR, and CD45) on HATMSC2 cells and HATMSC2-MVs from three independent experiments. Cells and MVs were stained with selected antibodies conjugated with fluorochromes. Blue filled histograms correspond to HATMSC2 cells and HATMSC2-MVs labeled with defined antibodies, and empty histograms represent the isotype controls. HATMSC2-MVs—microvesicles derived from immortalized human mesenchymal stem cells of adipose tissue origin.

The HATMSC2 cells and HATMSC2-MVs were tested for the presence of MSC markers CD73, CD90, CD105, the HLA ABC and HLA DR antigens, and the leukocyte marker CD45. The analysis confirmed the presence of CD73, CD90, and CD105 on the surface of HATMSC2 cells. The cells were also positive for the HLA ABC antigen and negative for the HLA DR antigen and for the pan-leukocyte antigen CD45 [16]. Importantly, HATMSC2-MVs expressed surface markers typical for MSCs, including CD73 (50.50 ± 2.18% of the population), CD90 (90.67 ± 5.36% of the population), CD105 (45.32 ± 3.24% of the population), and HLA ABC (88.20 ± 4.61% of the population). HATMSC2-MVs did not express HLA DR or CD45 (Figure 2).

### 2.3. Internalization of HATMSC2-MVs into Ovarian Cancer Cells

**Figure 3:**
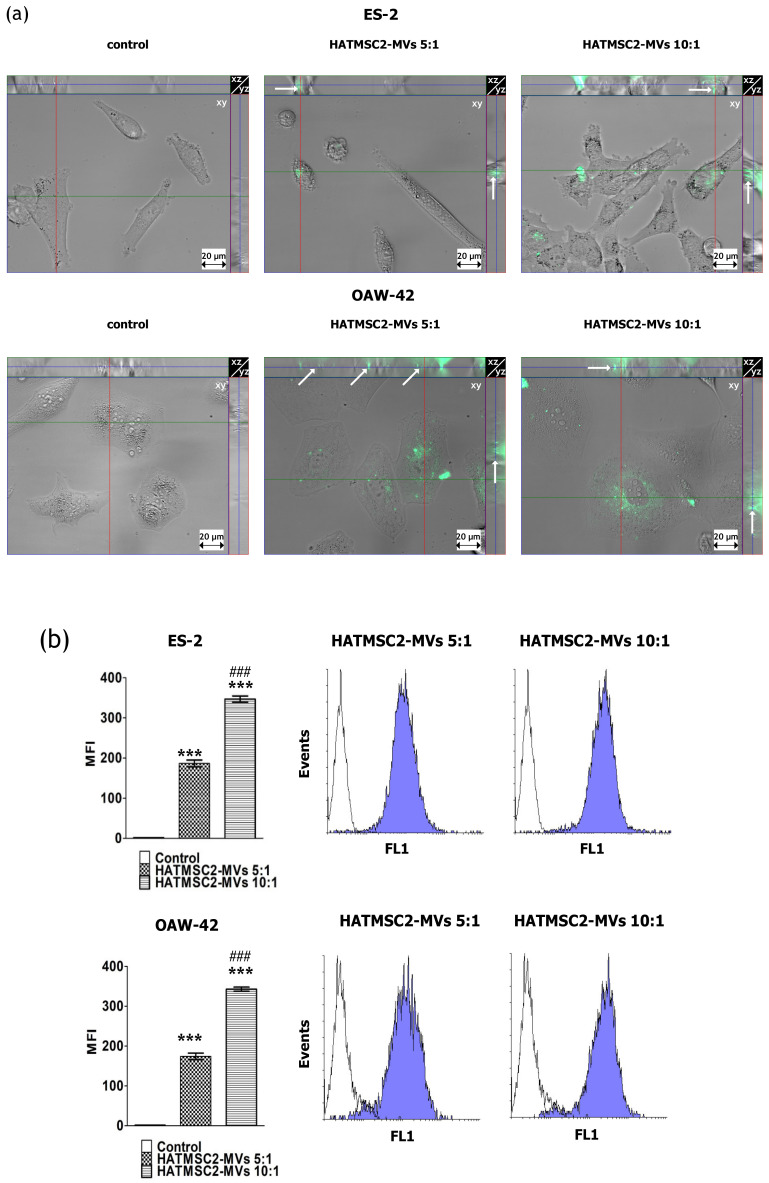
Internalization of HATMSC2-MVs into ovarian cancer cells. (**a**) Images from three-dimensional microscopic analysis of HATMSC2-MV internalization into ES-2 and OAW-42 cells at different ratios. The images were taken using an inverted microscope after 24 h of incubation with fluorescently-labeled HATMSC2-MVs (scale bar: 20 µm). A set of representative orthogonal slices is shown. Each image in a group consists of a large middle segment that represents the midpoint of the Z-stack in the *xy* plane; a narrow top segment that represents the *xz* plane; and a narrow segment on the right that represents the *yz* plane. The arrows point to MVs that have been taken up into the cell. (**b**) Bottom left panel, the bar graph represents the mean fluorescence intensity (MFI) of ES-2 cells treated with fluorescently labeled HATMSC2-MVs at different ratios. Untreated cells without MVs served as a control. Right panel, flow cytometry analysis of HATMSC2-MV internalization. Empty histograms represent the control for untreated cells, and blue filled histograms show the green fluorescence of ovarian cancer cells ES-2 and OAW-42 after HATMSC2-MV internalization at different ratios. The data represent mean ± SEM values from three independent experiments performed in duplicate. *** *p* < 0.001 calculated vs. control, ### *p* < 0.001 calculated vs. the HATMSC2-MVs 5:1 treatment. HATMSC2-MVs—microvesicles derived from immortalized human mesenchymal stem cells of adipose tissue origin.

The internalization of fluorescently-labeled HATMSC2-MVs into ovarian cancer cell lines was analyzed using three-dimensional microscopic imaging. HATMSC2-MV co-culture with target cells (ES-2 and OAW-42 cell lines) for 24 h resulted in the incorporation of the MVs into ES-2 and OAW-42 cells, as shown by green fluorescence (DiO) expression in the cytoplasm of the target cells across the Z-stack slices (Figure 3a).

Furthermore, the uptake of HATMSC2-MVs by ovarian cancer cells was confirmed by an increase in mean fluorescence intensity (MFI), as analyzed using flow cytometry. The results showed a significant increase in MFI in the ES-2 and OAW-42 cell lines treated with HATMSC2-MVs for both the ratios of 5:1 and 10:1 (the number of MVs to one target cell) compared to the control groups (*p* < 0.001). Moreover, this effect was dose-dependent, and significant differences between the ratios 5:1 and 10:1 (*p* < 0.001) were observed (Figure 3b).

### 2.4. Anti-Proliferative Activity of HATMSC2-MVs

**Figure 4:**
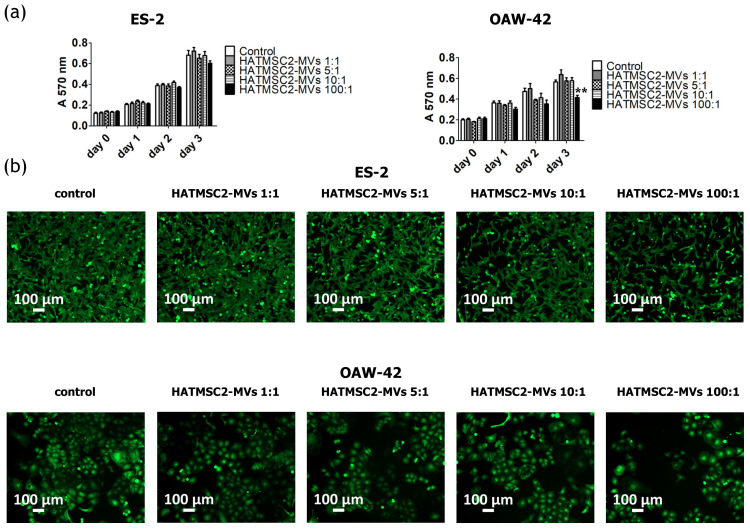
Effect of HATMSC2-MVs on the proliferation activity of ovarian cancer cells. (**a**) Proliferation activity of ES-2 and OAW-42 cells cultured in standard conditions was measured using an MTT assay on day 0, 1, 2, and 3 following treatment with HATMSC2-MVs at different ratios. Untreated cells without MVs served as a control. The data represent mean ± SEM values from four independent experiments performed in triplicate. ** *p* < 0.01 calculated vs. control on a given day. (**b**) Representative images from microscopic analysis of the morphology of ovarian cancer cells treated with HATMSC2-MVs at different ratios. ES-2 and OAW-42 cells were co-incubated with HATMSC2-MVs for 72 h. Afterwards, the cells were stained with Calcein AM and images were taken using an inverted microscope (scale bar: 100 µm). HATMSC2-MVs—microvesicles derived from immortalized human mesenchymal stem cells of adipose tissue origin.

The anti-proliferative activity of HATMSC2-MVs was analyzed using the MTT assay. ES-2 and OAW-42 cells were treated with MVs at four different ratios: 1:1, 5:1, 10:1, and 100:1. The HATMSC-MV treatment caused a significant decrease in OAW-42 cell proliferation on day 3 (*p* < 0.01) at a ratio of 100:1 (Figure 4a). The anti-proliferative activity of the MVs used at a ratio of 100:1 in OAW-42 cells on day 3 was also shown on a microscopic images of calcein-stained ovarian cancer cells (Figure 4b). 

### 2.5. Effect of HATMSC2-MVs on Cell Cycle Progression

**Figure 5:**
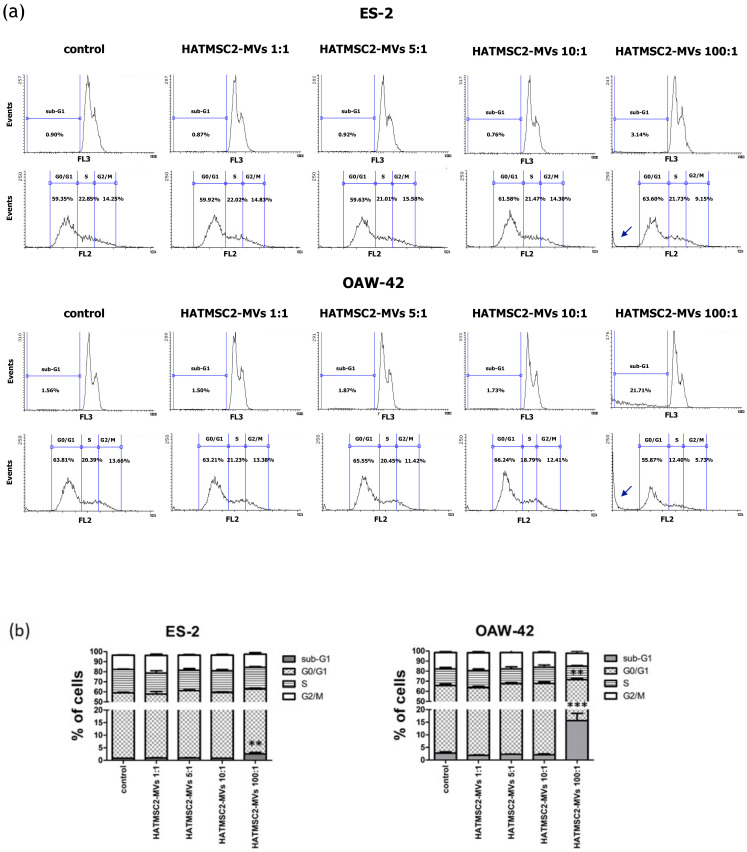
Effect of HATMSC2-MVs on the cell cycle progression of ovarian cancer cells. (**a**) Representative flow cytometry histograms showing cell cycle progression in ES-2 and OAW-42 cells treated with HATMSC2-MVs at different ratios. Untreated cells without MVs served as a control. Arrows represent the increased peaks in the sub-G1 phase of the cell cycle. (**b**) Percentages of cells in the sub-G1, G0/G1, S, and G2/M phases were determined using Flowing Software 2. The data represent mean ± SEM values from three independent experiments performed in duplicate. *** *p* < 0.001, ** *p* < 0.01 calculated vs. control. HATMSC2-MVs—microvesicles derived from immortalized human mesenchymal stem cells of adipose tissue origin

The effect of HATMSC2-MVs on cell cycle progression was tested using flow cytometry analysis of ES-2 and OAW-42 cells stained with propidium iodide. We observed an increase in the percentage of cells in the sub-G1 phase (dead cells) in the samples treated with the MV ratio of 100:1 in ES-2 cells, compared to the control group (mean 2.57 ± 0.54% vs. 0.79 ± 0.05%; *p* < 0.01). Similarly, in OAW-42 cells treated with the MVs ratio of 100:1, the percentage of cells in the sub-G1 phase increased to 15.66 ± 2.86% compared to 2.74 ± 0.48% in control group (*p* < 0.001). Moreover, in OAW-42 cells treated with an MVs ratio of 100:1, the percentage of cells in the G0/G1 phase decreased from 63.06 ± 1.49% in the control group to 55.87 ± 1.37% in the test group (*p* < 0.01), (Figure 5).

### 2.6. Proapoptotic Activity of HATMSC2-MVs

**Figure 6:**
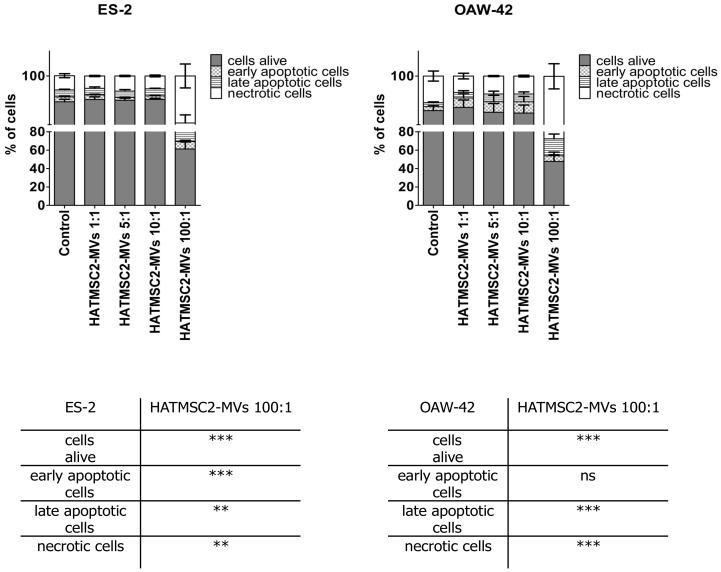
Quantification of cell viability after treatment with HATMSC2-MVs for 72 h at different ratios, determined using flow cytometric analysis of the apoptotic and necrotic cells via the double-staining of ES-2 and OAW-42 cells with propidium iodide and Annexin V. The percentages of alive, early apoptotic, late apoptotic, and necrotic cells were determined using Flowing Software 2. The data represent mean ± SEM values from five independent experiments performed in duplicate. *** *p* < 0.001, ** *p* < 0.01 calculated vs. control, ns: non-significant results. HATMSC2-MVs—microvesicles derived from immortalized human mesenchymal stem cells of adipose tissue origin.

We examined the impact of HATMSC2-MVs on ovarian cancer cell survival. Cell death processes, such as apoptosis and necrosis, were assessed using flow cytometry after 72 h of co-culture of HATMSC2-MVs with ES-2 and OAW-42 cells at the ratios of 1:1, 5:1, 10:1, and 100:1. Untreated cells served as a control. The obtained results revealed that the HATMSC2-MVs treatment affected cell viability depending on the ratio of HATMSC2-MVs and cancer cells. The ratio of MVs 100:1 had the greatest impact on cell viability, both in the ES-2 and OAW-42 cells (Figure 6). 

In ES-2 cells, the percentage of live cells treated with HATMSC2-MVs decreased to 61.23 ± 7.71% for the ratio of 100:1 vs. control (89.65 ± 0.99%; *p* < 0.001). The average percentage of early apoptotic cells increased to 8.22 ± 1.32% vs. control 1.95 ± 0.41%, (*p* < 0.001), whereas the average percentage of late apoptotic cells increased to 11.75 ± 3.22% vs. control 2.79 ± 0.32%, (*p* < 0.01). The average percentage of ES-2 necrotic cells increased to 18.81 ± 4.79% vs. control 5.72 ± 0.81%, (*p* < 0.01).

In OAW-42 cells, the percentage of live cells treated with HATMSC2-MVs decreased to 47.78 ± 10.11% for the ratio of 100:1 vs. control (86.17 ± 2.12%, *p* < 0.001). The average percentage of late apoptotic cells increased to 18.53 ± 5.17% vs. control 1.57 ± 0.34%, (*p* < 0.001). The average percentage of OAW-42 necrotic cells increased to 27.51 ± 5.04% vs. control 10.70 ± 2.02%, (*p* < 0.001).

### 2.7. Effect of HATMSC2-MVs on the Phenotype of Ovarian Cancer Cell Lines

**Figure 7:**
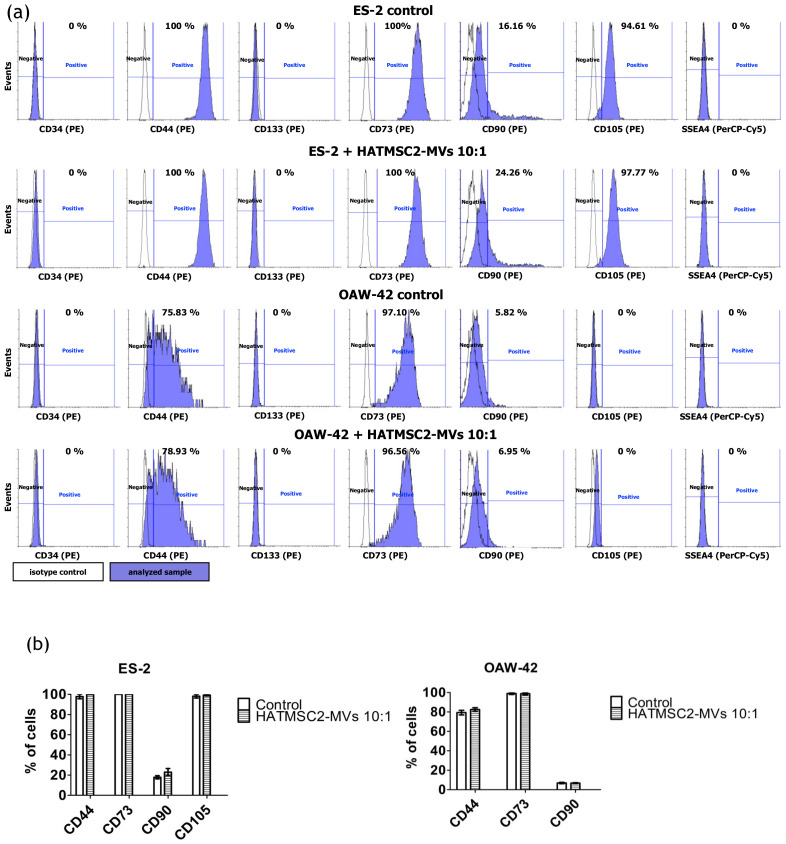
Characteristics of human ovarian cancer cell lines before and after HATMSC2-MV treatment at different ratios. (**a**) Representative histograms of flow cytometry analysis for surface markers (CD34, CD44, CD133, SSEA4, CD73, CD90, and CD105) on the ES-2 and OAW-42 cell lines. The cells were stained with selected antibodies conjugated with fluorochromes. Blue filled histograms correspond to ES-2 and OAW-42 cells, labeled with defined antibodies, and empty histograms represent the isotype controls. (**b**) The percentages of cells positive for selected markers were determined using Flowing Software 2. The data represent mean ± SEM values from three independent experiments. HATMSC2-MVs—microvesicles derived from immortalized human mesenchymal stem cells of adipose tissue origin.

To determine the effect of HATMSC2 -MVs on the phenotype of ovarian cancer cell lines treated at the ratios of 10:1 and 100:1, we tested the presence of the CD34, CD44, CD133, SSEA4, CD73, CD90, and CD105 markers using flow cytometry. The results showed that both cell lines, ES-2 and OAW-42, were positive for the adhesion molecule CD44 (Figure 7). However, the expression of the CD44 marker was higher (97.80% ± 2.20% of the population) for the ES-2 cells compared to OAW-42 cells (78.36% ± 3.20% of the population). Both ES-2 and OAW-42 cell lines were negative for CD34 and CD133 and for the pluripotency-related marker SSEA4. An analysis of the expression of MSC markers showed that both ES-2 and OAW-42 were positive for CD73 and CD90, whereas the CD105 marker was detected in ES-2 cells (98.20% ± 1.79% of the population), but not in OAW-42 cells (Figure 7a). The HATMSC2-MVs treatment did not affect the phenotype of ES-2 and OAW-42 cells (Figure 7b). 

### 2.8. Effect of HATMSC2-MVs on the Secretion Profile of Ovarian Cancer Cell Lines

**Figure 8:**
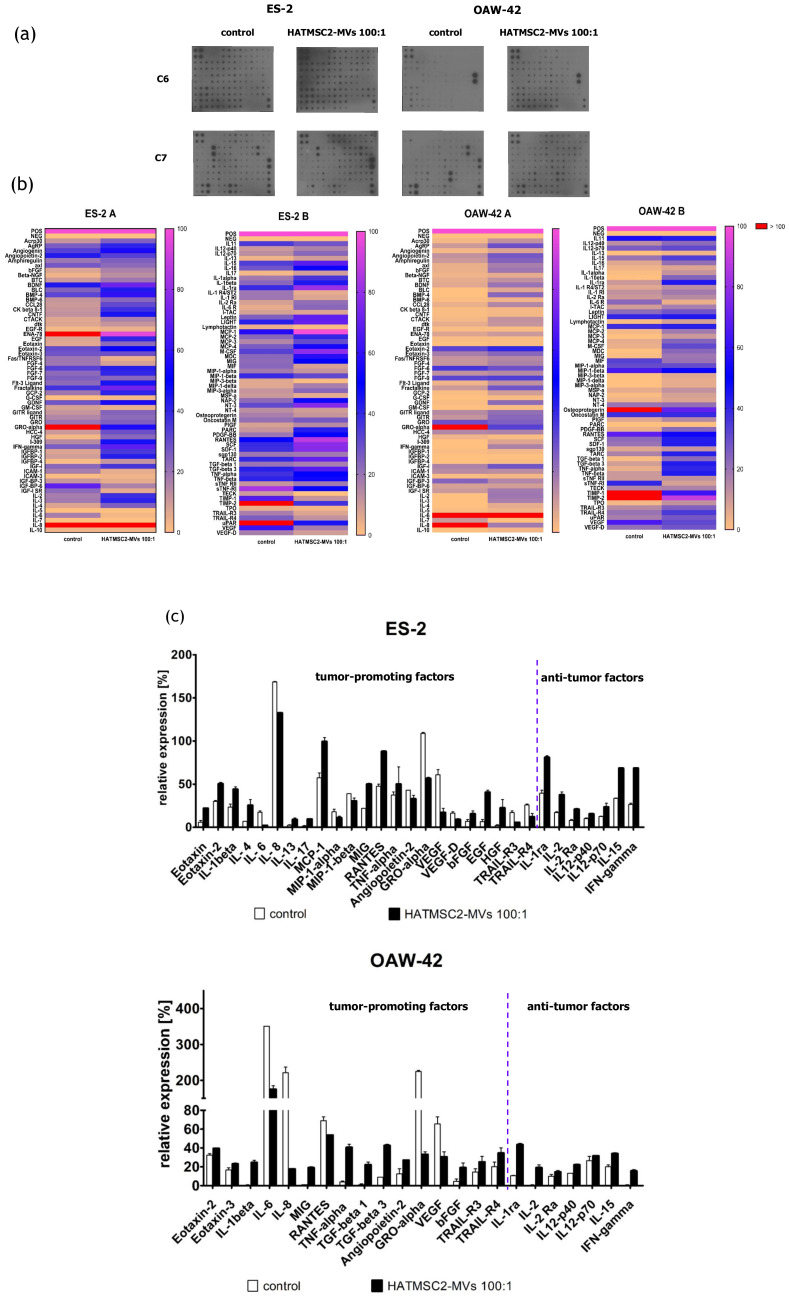
Effect of HATMSC2-MVs on the secretion profiles of ovarian cancer cell lines. (**a**) Scans of representative antibody arrays for ES-2 and OAW-42 supernatants. Untreated cells served as a control. The signal intensity for each antibody spot is proportional to the relative concentration of the antigen in the sample. (**b**) Heat map of cytokine levels for the ES-2 and OAW-42 supernatants; magenta and yellow indicate higher and lower expression limits, respectively. Outstanding values (above 100% of positive control) are depicted in red. Data are normalized to the internal positive control spots, which are consistent between the arrays and represent 100%. The data represent the mean from a duplicate assessment. (**c**) Column graph representing selected proteins (equal to or above 10% of the positive control). The data are presented as mean ± SEM values from a duplicate assessment. HATMSC2-MVs—microvesicles derived from immortalized human mesenchymal stem cells of adipose tissue origin.

The effect of HATMSC2-MVs on the biological properties of ovarian cancer cells was determined in all experiments at different ratios of MVs to cancer cells (1:1, 5:1, 10:1 and 100:1). However, the best effect was seen when a ratio of 100:1 was used. Therefore, to determine the effect of HATMSC2-MVs on the secretion profile of the ES-2 and OAW-42 cell lines, only the ratio of 100:1 was used. The secretion profile was evaluated using a human cytokine antibody array (Figure 8a). Most of the 120 cytokines and trophic factors identified in this analysis affect cancer cells either by promoting cancer growth or through their anti-tumor properties. For the ES-2 cells treated with HATMSC2-MVs, among cancer-promoting cytokines and trophic factors, a decrease was observed for angiogenesis-related cytokines, such as growth related oncogene-alpha (GRO-alpha) (by 53%), angiopoietin 2 (by 10%), VEGF (by 44%), and VEGFD (by 8%), and for the pro-angiogenic and pro-inflammatory cytokines IL-6 (by 15%), IL-8 (by 36%), MIP-1α (by 7%), and MIP-1β (by 8%). The levels of apoptosis-related TRAIL-R3 and TRAIL-R4 also decreased (by 10% and 14%, respectively). However, an increase was observed in the levels of the pro-inflammatory cytokines IL-1α (by 18%), IL-1β (by 21%), MCP-1 (by 43%), MIG (by 28%), TNFα (by 13%), IL-13 (by 6%), eotaxin (by 16%), and eotaxin-2 (by 21%), and growth factors bFGF (by 9%), EGF (by 33%), and HGF (by 21%). On the other hand, we observed an increase in the levels of several anti-cancer cytokines, such as IL-1 receptor antagonist (IL-1ra) (by 42%), IL-2 by (21%), IL-2 receptor alpha chain (IL-2Ra) (by 13%), IL-12 (by 6% and 10% for the p40 and p70 subunits, respectively), IL-15 (by 34%), and IFN-γ (by 43%). Nevertheless, the overall number of expressed cytokines in the ES-2 cells was 92; however, the expression of a majority of these cells differed before and after the HATMSC2-MV treatment (Figure 8b).

For the OAW-42 cells, the overall number of expressed cytokines increased from 58 in the control group to 87 after the MV treatment. However, most of these cytokines did not show a major difference in expression between the control and test groups. Among the tumor-promoting cytokines, we observed a decrease in the levels of the pro-angiogenic and pro-inflammatory cytokines GRO-alpha (by 192%), VEGF (by 34%), IL-6 (by 176%), IL-8 (by 204%), and RANTES (by 16%), and an increase in other cytokines, such as eotaxin-2 (by 8%), eotaxin-3 (by 6%), IL-1β (by 24%), MIG (by 18%), TNFα (by 36%), TGF-beta1 (by 21%), and -beta3 (by 34%), bFGF (by 15%), TRAIL-R3 (by 11%), and TRAIL-R4 (by 15%). An increase in the levels of anti-cancer cytokines was similar as observed for the ES-2 cells. All data were presented as a heat map (Figure 8b); selected cytokines which exhibited the largest differences between the treated cells and the control group were additionally shown on a column graph (Figure 8c).

## 3. Discussion

The paracrine activity of cells via EVs is an important link in cell-to-cell communication. Recent research has shown that EVs derived from MSCs play an important role in tumor microenvironment. Tumor cells secrete EVs to reprogram the mesenchymal stem cells present in the tumor microenvironment. The reprogrammed MSCs release exosomes that affect other cells in the tumor niche, such as fibroblasts, endothelial cells, and immune cells, inducing their pro-tumorigenic activity [17]. However, the effect of MVs derived from outside the tumor microenvironment, e.g., from the MSCs of adipose tissue origin, on cancer cells is not well understood and still debatable. The purpose of this study was to analyze the biological behavior of two histologically different ovarian cancer cell lines, ES-2 and OAW-42, in response to HATMSC2-MV treatment. In this study, we investigated whether MVs derived from human immortalized MSCs of adipose tissue origin may represent a new form of supportive therapy in ovarian cancer treatment. Flow cytometry and microscopic analysis confirmed the internalization of HATMSC2-MVs into target cells. Moreover, in all functional experiments, we used untouched MVs, but not the MVs lysate tested by different research groups [14]. We showed that treatment with HATMSC2-MVs gradually decreased the proliferation of ES-2 and OAW-42 cells, depending on the dose; however, a significant effect was observed on day 3, only for the OAW-42 cell line, when the highest ratio of HATMSC2-MVs of 100:1 (100 MVs per cell) was used. Similar results were obtained by Reza et al. [14], who reported an anti-proliferative and pro-apoptotic effect of ATMSC exosomes on ovarian cancer cells. The main mechanism involved in the action of the exosomes was the transfer of different miRNAs into the recipient cells. However, Reza et al. used protease or RNase-digested exosomes. The anti-proliferative activity of MVs derived from BM-MSCs was also confirmed in vitro on the SKOV3 cell line and in vivo through an intra-tumor injection of MVs into an established tumor generated by a subcutaneous injection of these cells into SCID mice [18]. On the other hand, Dong et al. [19] showed that EVs derived from MSCs isolated from the umbilical cord increased the proliferation of lung adenocarcinoma cells by transporting miR-410 to target cells.

Further experiments involving an analysis of cell cycle progression and cell death processes, such as apoptosis and necrosis, confirmed that HATMSC2-MV treatment affected cancer cell viability. The cell cycle analysis showed that treatment with HATMSC2-MVs, at the ratio of 100:1, significantly increased the percentage of cells of both cell lines in the sub-G1 phase compared to control; however, the increase of OAW-42 cells in the sub-G1 phase was significantly higher compared to the ES-2 cell line. The increased number of cells in the sub-G1 phase suggests that both ovarian cancer cell lines underwent cell death via apoptosis. A similar pro-apoptotic effect of the bioactive factors derived from MSCs isolated from human Wharton’s jelly and applied in the form of a conditioned medium or Wharton’s jelly-MSCs lysate was observed in a study on the OVCAR3 and SCOV3 ovarian cancer cell lines, confirming the anti-cancer properties of the MSC secretome [20]. The pro-apoptotic effect of HATMSC2-MVs on the examined ovarian cancer cell lines was also confirmed through flow cytometry analysis of cell death processes, distinguishing between apoptosis and necrosis. When HATMSC2-MVs were cultured with ovarian cancer cell lines at the ratio of 100:1, we observed a significant increase in the percentage of early and late apoptotic cells for the ES-2 cells, whereas in the OAW-42 cells, a substantial increase was observed for late apoptotic cells. Moreover, in both cells lines, HATMSC2-MVs significantly increased the percentage of necrotic cells. These results suggest that HATMSC2-MVs at a ratio of 100:1 induce mechanisms governing ovarian cancer cell death via both apoptosis and necrosis. Studies on the anti-cancer properties of the MSC secretome report that co-culture of MSCs of different tissue origin with ovarian cancer cell lines increases apoptosis with varying effects [21]. Interestingly, the percentage of apoptotic cells was higher when the supernatant derived from AT-MSCs was applied compared to the supernatants derived from BM-MSCs and UC-MSCs [21].

Additionally, we assessed the secretion profile of ovarian cancer cell lines and the effect of HATMSC2-MVs on the presence of the produced cytokines and trophic factors with different functions; one set of bioactive factors is known to promote cancer cells growth and metastasis, and the second set of cytokines is associated with anti-tumor properties. The differences in the secretion profiles of the examined ovarian cancer cell lines correlated with the histological type of the tumor. ES-2 cells were derived from clear cell carcinoma, with a good prognosis for the patient when diagnosed at an early stage of the disease and poor survival when diagnosed at an advanced stage, because this type of ovarian cancer is often more resistant to chemotherapy than serous cystadenocarcinoma, represented by the OAW-42 cell line [22]. The presence of cancer-promoting cytokines and chemokines, such as IL-6, IL-8, GRO-alpha, MIP-1α, MIP-1β, angiopoetin-2, and VEGF, which are associated with tumor growth, metastatic properties, and a poor prognosis, was detected in the supernatants collected from both ES-2 cells and OAW-42 cells. The application of HATMSC2-MVs resulted in a substantial decrease in IL-6, IL-8, GRO-alpha, and VEGF secretion in both cell lines. IL-8 is a pleiotropic chemokine with a dual function, which acts as a chemoattractant for neutrophils, inducing innate immune responses, whereas in the ovarian cancer environment, it contributes to the pro-survival activity of tumor cells and resistance to chemotherapy. A high production of IL-8 correlates with faster proliferation and increases the potential of angiogenesis, adhesion, and invasion of platinum sensitive (PEA1 and PEO14) and platinum resistant (PEA2 and PEO23) cell lines [23]. Browne et al. demonstrated a significant increase in the expression of IL-8 in specimens of the serous type of ovarian cancer compared to clear cancer ovarian carcinoma tissue [24]. The HATMSC2-MVs markedly inhibit IL-8 production in both examined cell lines in vitro. This effect can be used as a potential supportive therapy for ovarian cancer treatment. IL-6 was present in the OAW-42 culture supernatant at a very high level, in contrast to the ES-2 cells. It is well known that IL-6 plays a crucial role in the stimulation of inflammatory cytokine production, tumor angiogenesis, cell proliferation, and tumor macrophage infiltration [25]. A high production of IL-6 by ovarian cancer cells contributes to tumor progression and correlates with a poor prognosis [26]. The HATMSC2-MVs inhibit the activity of IL-6, and in conjunction with a decreased level of IL-8, may exert suppressive effects on the ovarian cancer cell line. GRO-alpha and VEGF were produced by ES-2 and OAW-42 cells; however, the GRO-alpha level markedly exceeded the VEGF level. Both growth factors, GRO-alpha and VEGF, are important for tumor growth and metastasis, especially in terms of supporting cancer angiogenesis. The diverse production of cytokines and growth factors by ovarian cancer cells is associated with the biological activity of cancer cells and may affect tumor progression, as reported in a study performed simultaneously on a set of 120 cytokines in ovarian cancer ascites [25]. Our study determined that HATMSC2-MVs substantially reduced the secretion of GRO-alpha and VEGF in both cell lines. Numerous growth factors and cytokines, including those assessed in our study, such as IL-6, IL-8, MCP-1, RANTES, GRO-alpha, and VEGF, are involved in promoting tumor growth and ovarian cancer cell aggressiveness. Therefore, characterizing cytokine secretion may provide information on the functional profile of cancer cells. This may help to create targeted therapy for ovarian cancer, in which angiogenesis is inhibited by a blockage of NF-κB, suppressing VEGF and IL-8 activity [27], or by targeting CXCR2, the key receptor for the GRO-alpha and IL-8 chemokine activity [28]. RANTES (CCL5) level decreased only in OAW-42 cells after the HATMSC2-MVs treatment. RANTES is involved in trafficking immune cells into the inflammation site and acts as a co-activator of T cells promoting the polarization of the immune response towards the Th1 profile. In the ovarian cancer microenvironment, RANTES acts through paracrine or autocrine signaling to promote tumor cell migration, invasion, and metastasis [29]. In contrast, MCP-1 was detected in supernatants collected from ES-2 cells, and its level increased after the HATMSC2-MV treatment. The main function of MCP-1 in the tumor microenvironment is to attract tumor-associated monocytes (TAMs) [30]. Research performed by Furukawa et al. [30] demonstrated that the MCP-1 chemokine promoted the invasion and adhesion of the ovarian cancer cell line SKOV3, contributing to the progression of tumors. Tumor necrosis factor-related apoptosis-inducing ligand (TRAIL)-R3 and –R4 are known as the negative regulators of TRAIL-mediated apoptosis in cancer cells [31,32,33]. The internalization of HATMSC2-MVs by the examined cell lines exerts a different effect on ovarian cancer cells, and decrease the level of TRAIL-R3 and TRAIL-R4 in ES-2 cells and increase their level in OAW-42 cell lines. The downregulation of TRAIL-R3 and TRAIL-R4 is associated with an increased level of early apoptotic cells in the ES-2 cell line treated with the 100:1 ratio. A very recent study, performed on a murine xenograft model, documented that the EVs isolated from the TRAIL expressing cell line 293T in combination with cyclin-dependent kinase inhibitor (dinaciclib) successfully inhibited the growth of human lung cancer cell lines NCI-H727 and A549 and the human breast adenocarcinoma cell line MDAMB231 by inducing apoptosis [34].

The HATMSC2-MVs used in this study affect both histologically different cell lines, the ES-2 cells and the OAW-42 cells, by increasing the production of tumor-suppressive cytokines, such as IL-1ra, IL-2, IL-2Ra, IL-12-p40, IL12-p70, IL-15, and IFN-γ. Studies that used bioactive factors released to the culture medium from Wharton’s jelly MSCs led to a similar inhibition of the proliferation of the ovarian cancer cell line OVCAR3 through a decreased expression of oncogenic cytokines and growth factors and an increased expression of anti-tumor related cytokines [35]. The anti-inflammatory properties of IL-1ra, a naturally occurring inhibitor to IL-1, contribute to tumor growth inhibition by competitive binding to IL-1 receptors blocking cancer-promoting activity of IL-1 [36]. The anti-proliferative effect of ovarian cancer cell lines can be also supported by an increased production of IFN-γ following the exposure of cells to HATMSC2-MVs, as documented in both examined cell lines. It has been reported that IFN-α and IFN-γ, applied in combination with IL-4 fused to Pseudomonas exotoxin, inhibit tumor growth in an experimental mouse model of human ovarian cancer. The anti-tumor effect was accomplished by the activation of the IFN signaling pathways and the subsequent activation of molecules inducing apoptotic cell death [37]. Characterization of a wide range of tumor-promoting factors and anti-tumor cytokines after ovarian cancer cell expose to HATMSC2-MVs provides information on how they affect the production of functional cytokines and shed light on the mechanism altering the behavior of ovarian cancer cells in response to MV treatment.

Consequently, we characterized the ovarian cancer cell lines ES-2 and OAW-42 for the presence of CSC and MSC markers. The results showed that the ES-2 and OAW-42 cells were positive for CD44 and negative for CD133, and that the application of HATMSC2-MVs had no effect on the expression of these markers. Similar results were reported by Tudrej et al., who showed that CD44 expression was higher for ES-2 cells compared to OAW-42 cell lines [38]. They found a small subpopulation of ES-2 cells positive for the CD133 marker expression (around 0.2%). However, we did not observe any CD133 positive cells in our study. To our best knowledge, the expression of specific MSC markers on ovarian cancer cell lines has been studied in the form of a single MSC marker as a potential therapeutic target [39,40,41,42], whereas a complete analysis of MSC markers has been performed in a limited number of studies concerning the biological activity of ovarian cancer cell lines [21]. Our results revealed that both ES-2 and OAW-42 cells were strongly positive for CD73, and that HATMSC2-MVs had no impact on CD73 expression. CD73, also known as cell surface nucleotidase, is an immunosuppressive enzyme involved in tumor progression and metastasis, and its expression is associated with a poor prognosis for high-grade serous ovarian cancer [42]. The functional inhibition of CD73 via either a chemical compound or a neutralizing antibody reduced the tumorigenesis of primary high-grade serous epithelial ovarian cancer cells [41]. In contrast, CD90 was present on a limited population of both examined cell lines, and co-culture of ES-2 and OAW-42 cells with HATMSC2-MVs at a ratio of 1:10 did not increase the expression of this marker. It was reported previously that the overexpression of CD90 inhibited the sphere-forming ability of SKOV3 cell lines and increased cell apoptosis. These studies also suggest that CD90 may decrease cell growth through a downregulation of the expression of other CSC markers, including CD133 and CD24 [40]. Interestingly, the CD105 molecule was detected only on poorly-differentiated ES-2 cells, but not on the better-differentiated OAW-42 cells. This finding confirmed the mesenchymal phenotype of ES-2 cells, which is associated with increased aggressiveness and metastatic potential. The HATMSC2-MVs have no marked impact on CD105 expression in either of the cell lines. Studies on the biological role of CD105 in ovarian cancer revealed that high CD105, CD44, or CD106 expression was associated with drug resistance, an advanced stage of the disease, poor differentiation, and high rate of cancer relapse [43]. The downregulation of CD105 expression with a clinically relevant CD105-neutralizing mAb (TRC105) inhibited high-grade serous cancer metastasis, reduced ascites, and hampered the growth of abdominal tumor nodules in animal models of ovarian cancer [39].

A systematic review, introducing the impact of experimental anti-tumor cellular therapies involving MSCs of different human tissue origin, also highlights the possibility to use MSC secretome, in the form of a conditioned medium or EVs, as a cell-free therapy to inhibit cancer growth [44]. Thus, MVs may serve as a carrier for the delivery of therapeutic agents to target cells. A modification of primary MSCs for the secretion of inhibitory growth factors and pro-apoptotic factors may be employed to prepare the MVs carrying the pro-apoptotic signal and transport them to target ovarian cancer cells. Thus, MVs may be applied as a supportive therapy to enhance the therapeutic effect of chemotherapy, especially for multidrug resistant cancers. 

In conclusion, we confirmed an effective transfer of HATMSC2-MVs into target ovarian cancer cells, which affected the biological behavior of these cells. Our results revealed that HATMSC2-MVs inhibit tumor cell proliferation in the two histologically distinct ovarian cancer cell lines via different pathways, apoptosis and/or necrosis. This phenomenon, with high likelihood, is related to the secretion of the different anti-tumor factors by the ES-2 (representing poorly differentiated ovarian clear cell carcinoma), and OAW-42 (representing ovarian cystadenocarcinoma) cell lines treated with HATMSC2-MVs. However, further studies are needed to determine the possible mechanisms involved in HATMSC2-MV-mediated effect on target cells, as well as to validate their anti-tumorigenic potential with respect to cancer cells isolated from human tissues. Therefore, understanding the mechanisms involved in the bilateral interaction between the MVs and ovarian cancer cells may be help to design new treatment modalities for an effective anti-tumor cell-free therapy.

## 4. Materials and Methods

### 4.1. Cell Culture

The ES-2 cell line was purchased from ATCC (American Type Culture Collection, Manassas, VA, USA) (catalog number: CRL-1978™). The cells were cultured in the DMEM and OptiMEM GlutaMax media, mixed in equal proportions. The DMEM medium was supplemented with 10% FBS (Gibco, Thermo Scientific, Carlsbad, CA, USA), a 1% penicillin/streptomycin solution (Gibco, Thermo Scientific, Carlsbad, CA, USA) and L-glutamine (Gibco, Thermo Scientific, Carlsbad, CA, USA). The OptiMEM GlutaMax medium was supplemented with 3% FBS (Gibco, Thermo Scientific, Carlsbad, CA, USA) and a 1% penicillin/streptomycin solution (Gibco, Thermo Scientific, Carlsbad, CA, USA).

The OAW-42 cell line was purchased from ECACC (European Collection of Authenticated Cell Cultures, Salisbury, United Kingdom) (catalog number: 85073102). The cells were cultured in the same media conditions, mixed in equal proportions, and additionally supplemented with a 10 µg/mL insulin solution (Sigma-Aldrich, St. Louis, MO, USA).

All cells were cultured in standard conditions (21% O_2_, 5% CO_2_, 95% humidity, 37 °C temperature). Upon reaching 70–80% confluence, the cells were harvested with a 0.05% trypsin/0.02% EDTA (*w/v*) solution (IIET, Wroclaw, Poland) and seeded onto new culture flasks.

The human mesenchymal stem cell line HATMSC2 was established in our laboratory using the hTERT and pSV402 plasmids, as described in a previous study [16].

### 4.2. MV Isolation Using Sequential Centrifugation

MVs were isolated according to the well-established protocol developed in our laboratory [45] based on the procedure introduced in the previous study [46]. HATMSC2 cells were cultured in multi-layer cell culture flasks (Nunc TripleFlasks, Thermo Scientific, Carlsbad, CA, USA) using DMEM + 10% FBS until they reached 75% confluence. Next, the cells were cultured in serum-free media in hypoxic conditions (1% O_2_) for 48 h to enhance the release of MVs. The conditioned media collected from the HATMSC2 cultures were mixed to obtain a homogenous starting material before the isolation of MVs. In the next step, the conditioned media were centrifuged at 300× *g* for 10 min at 4 °C, and at 2000× *g* for 10 min at 4 °C, in order to remove cellular debris and apoptotic bodies. Subsequently, the supernatants were subjected to double centrifugation at 12,000× *g* for 30 min at 4 °C using a Sorvall LYNX 6000 ultracentrifuge (Thermo Scientific, Carlsbad, CA, USA) with an intermediate washing step in PBS (IIET, Wroclaw, Poland). The obtained MV pellets were resuspended in 150 μL of PBS and stored at −80 °C.

### 4.3. Analysis of MVs

The size distribution of MVs was measured with DLS (Malvern Zetasizer, Malvern, UK). The measurement was performed for 2 min at 25 °C. PBS was used to disperse the samples. Moreover, to confirm the size of the MVs, the samples were analyzed using transmission electron microscopy (TEM). The PBS-suspended MVs were placed on a carbon-coated copper grid (400 mesh) and incubated for 1 min, and the excess liquid was removed with filter paper. Next, the samples were stained with 2% uranyl acetate, dried, and examined with a transmission electron microscope (JEOL, Peabody, MA, USA) at 80 kV. The number of MVs was calculated using a BD Fortessa Flow Cytometer (BD Biosciences, San Jose, CA, USA) and fluorescent counting beads (CountBright™ Absolute Counting Beads for flow cytometry, Thermo Scientific, Carlsbad, CA, USA). Prior to analysis, 10 µL of the MV sample was diluted in PBS to a final volume of 300 µL, after which 50 µL of counting beads were added. The threshold for the forward scatter (FSC) was set at 200. To determine the number of MVs in the samples, 5000 counting beads were collected using a BD Fortessa Flow Cytometer (BD Biosciences, San Jose, CA, USA). The data were analyzed using the BD FACSDiva Software (BD Biosciences, San Jose, CA, USA). The number of MVs was calculated according to the CountBright™ Absolute Counting Beads manufacturer’s instructions, using the ratio of MV events and the number of counting bead events. The protein concentration of MVs was determined with a Bradford assay (Thermo Scientific, Carlsbad, CA, USA) according to the vendor’s instructions. The MV samples or the BSA standard were briefly incubated with the Bradford reagent for 5 min on a 96-well plate. The absorbance was measured with a Synergy H4 plate reader (Biotek, Winooski, VT, USA) at 595 nm.

### 4.4. Flow Cytometry Analysis of HATMSC2 Cells and HATMSC2-MVs

HATMSC2 cells were detached using the trypsin/EDTA solution and incubated with PE-conjugated antibodies specific for the human CD73 (clone AD2), CD90 (clone 5E10), CD105 (clone 266), HLA ABC (clone G46-2.6), HLA DR (clone G46-6) (BD Biosciences, San Jose, CA, USA), and FITC-conjugated CD45 antibody (clone 2D1) (BD Biosciences, San Jose, CA, USA) and with the appropriate isotype controls (BD Biosciences, San Jose, CA, USA) for 30 min at 4 °C. Afterwards, the labeled cells were washed with PBS (IIET, Wroclaw, Poland) and analyzed using a FACSCalibur flow cytometer (BD Biosciences, San Jose, CA, USA). The obtained data were processed using the CellQuest software (BD Biosciences, San Jose, CA, USA). The histograms were created using Flowing Software 2. The surface markers of the MVs were analyzed using a BD Fortessa Flow Cytometer (BD Biosciences, San Jose, CA, USA) after staining with specific fluorophore-conjugated antibodies. MVs suspended in PBS were incubated with PE-conjugated antibodies specific for human CD73, CD90, and CD105, HLA ABC, HLA DR, and FITC-conjugated antibody for CD45 and with the appropriate isotype controls for 30 min at 4 °C. The percentage of positive MVs was calculated using the BD FACSDiva Software (BD Biosciences, San Jose, CA, USA).

### 4.5. Internalization of MVs

ES-2 and OAW-42 cells were seeded into a Lab-Tek II Chambered # 1.5 Coverglass system (Nalge Nunc International, Naperville, IL, USA) at a density of 15 × 10^3^ cells per chamber. Fluorescence staining of the MVs was performed, as established in our recent study [45]. After washing with PBS, the MVs were resuspended in the DMEM + 10% FBS and OptiMEM GlutaMax + 3% FBS media (mixed in equal proportions), and added to the cells at a ratio of 5:1 (5 MVs per cell) and 10:1 (10 MVs per cell). The cells were incubated with MVs for 24 h and washed twice with PBS prior to imaging. The internalization of the MVs into target cells was immediately analyzed at 37 °C using an Axio Observer inverted microscope equipped with a dry 63x objective (Zeiss, Gottingen, Germany). The labeled MVs were detected using an EGFP Filter set. Thirty Z-sections with a 0.6-μm interval were recorded simultaneously in the brightfield and fluorescence channel. Optical orthogonal sectioning was performed in order to visualize the internalization of the MVs. Images were obtained and processed using the Zen Blue Software (Zeiss, Gottingen, Germany). A similar analysis of EV internalization using fluorescence microscopy was previously described by Adamiak et. al. [47]. After 24 h of incubation with MVs, the cells were washed once with PBS, detached using the trypsin/EDTA solution, washed once more with PBS, and analyzed using flow cytometry with FACSCalibur (BD Biosciences, San Jose, CA, USA). The cells were detected using the FL1 channel (480 nm). The histograms were created using Flowing Software 2 (Perttu Terho, Turku Centre for Biotechnology, Finland).

### 4.6. Proliferation Activity

The proliferation activity of ES-2 and OAW-42 treated with HATMSC2-MVs was measured using the MTT assay. The cells were seeded on a 96-well plate at a concentration of 2 x 10^3^ cells/well in the DMEM + 10% FBS and OptiMEM GlutaMax + 3% FBS media (mixed in equal proportions); MVs at a ratio of 1:1 (1 MV per cell), 5:1 (5 MVs per cell), 10:1 (10 MVs per cell), and 100:1 (100 MVs per cell) were added to the cells. ES-2 and OAW-42 cells without MVs were used as a control. After 4 h, 24 h, 48 h, and 72 h, the absorbance of the formazan dye produced by living cells was measured using a Wallac 1420 Victor2 Microplate Reader (Perkin Elmer, Waltham, MA, USA) at 570 nm. After 72 h of co-incubation with HATMSC2-MVs, the ovarian cancer cells were stained using Calcein AM (Thermo Fisher, Carlsbad, USA). 100 µL of Calcein AM (1 µM solution) were added to each well. The cells were incubated for 15 min at room temperature. Images were obtained using an Axio Observer inverted microscope equipped with a dry 10x objective (Zeiss, Gottingen, Germany). The labeled cells were detected using an Alexa Fluor 488 Filter set. The images were processed with the Zen Blue software (Zeiss, Gottingen, Germany).

### 4.7. Cell Cycle Analysis

The cell cycle analysis was performed based on previously published method [20,48]. The cells were seeded on 24-well plates at a concentration of 12 × 10^3^ in the DMEM + 10% FBS and OptiMEM GlutaMax + 3% FBS media (mixed in equal proportions). MVs at a ratio of 1:1 (1 MV per cell), 5:1 (5 MVs per cell), 10:1 (10 MVs per cell), and 100:1 (100 MVs per cell) were added to the cells. ES-2 and OAW-42 cells without MVs were used as a control. After 72 h, the cells were detached using the trypsin/EDTA solution; the conditioned media were also collected and mixed with the respective cell suspension samples. The samples were centrifuged at 1400 rpm for 4 min at 4 °C. After the supernatant was removed, the cells were resuspended in ice-cold 70% ethanol and incubated for 30 min on ice at 4 °C. Afterwards, PBS Ca^2+^ Mg^2+^ + 2.5% FBS was added to the cells, and the samples were centrifuged at 1400 rpm for 5 min. This step was repeated twice. The cells were then resuspended in a solution of propidium iodide in PBS (50 µg/mL) and RNase (20 µg/mL) and incubated overnight at 4 °C. The cells were analyzed using a FACSCalibur flow cytometer (BD Biosciences, San Jose, CA, USA). The obtained data were analyzed using Flowing Software 2 (Perttu Terho, Turku Centre for Biotechnology, Finland).

### 4.8. Cell Viability and Apoptosis Analysis Using Flow Cytometry

In order to determine the effect of HATMSC2-MVs on the viability of the cells, an Annexin V Apoptosis Detection Kit (Thermo Scientific, Carlsbad, CA, USA) was used. ES-2 and OAW-42 cells were seeded in a 24-well plate at a density of 25 × 10^3^ in the DMEM + 10% FBS and OptiMEM GlutaMax + 3% FBS media (mixed in equal proportions). Before the analysis, the cells were treated with MVs at a ratio of 1:1 (1 MV per cell), 5:1 (5 MVs per cell), 10:1 (10 MVs per cell), and 100:1 (100 MVs per cell) for 72 h. ES-2 and OAW-42 cells without MVs were used as a negative control. After incubation with MVs, the cells were stained with Annexin V and propidium iodide according to the manufacturer’s recommendations. The cells were analyzed for live (Annexin V negative and propidium iodide negative), early apoptotic (Annexin V positive and propidium iodide negative), late apoptotic (Annexin V positive and propidium iodide positive), and necrotic cells (Annexin V negative and propidium iodide positive) using a FACSCalibur flow cytometer (BD Biosciences, San Jose, CA, USA). The analysis was performed using Flowing Software 2 (Perttu Terho, Turku Centre for Biotechnology, Finland).

### 4.9. Flow Cytometry Analysis of Ovarian Cancer Cell Lines

In order to determine the effect of HATMSC2-MVs on the phenotype of ovarian cancer cell lines, flow cytometry analysis was performed. ES-2 and OAW-42 cells were treated for 72 h with MVs at a ratio of 10:1. ES-2 and OAW-42 cells were seeded in a 6-well plate at a density of 6 x 10^4^ per well in the DMEM + 10% FBS and OptiMEM GlutaMax + 3% FBS media (mixed in equal proportions). MVs were added to the cells at a ratio of 10:1. ES-2 and OAW-42 cells without MVs were used as a control. After 72 h, the cells were washed with PBS, and the culture medium was replaced with DMEM without FBS. Following a subsequent 24 h of culture in DMEM without FBS, the cells were detached using the trypsin/EDTA solution and incubated with PE-conjugated antibodies specific for the human CD34 (clone 8G12), CD44 (clone 515), CD133 (clone W6B3C1), CD73 (clone AD2), CD90 (clone 5E10), and CD105 (clone 266) molecules and the PerCP-Cy5.5—SSEA4 antibody (clone MC813-70) (all from BD Biosciences, San Jose, CA, USA) and with the appropriate isotype controls (BD Biosciences, San Jose, CA, USA) for 30 min at 4 °C. Afterwards, the labeled cells were washed with PBS (IIET, Wroclaw, Poland) and analyzed using a FACSCalibur flow cytometer (BD Biosciences, San Jose, CA, USA). The obtained data were processed using the CellQuest software (BD Biosciences, San Jose, CA, USA). The histograms were created using Flowing Software 2 (Perttu Terho, Turku Centre for Biotechnology, Finland).

### 4.10. Secretion Profiles of Ovarian Cancer Cell Lines

In order to determine the effect of HATMSC2-MVs on the secretion profiles of ovarian cancer cell lines, a C-Series Human Cytokine Antibody Array C1000 (RayBio^®^, Norcross, GA, USA) was used. ES-2 and OAW-42 cells were seeded in a 6-well plate at a density of 6 × 10^4^ per well in the DMEM + 10% FBS and OptiMEM GlutaMax + 3% FBS media (mixed in equal proportions). MVs were added to the cells at a ratio of 100:1. The ES-2 and OAW-42 cells without MVs were used as a control. After 72 h, the cells were washed with PBS, and the culture medium was replaced with DMEM without FBS. Following the subsequent 24 h of culture in DMEM without FBS, the conditioned medium was collected and centrifuged for 10 min at 300× *g* to remove cellular debris, and the cells were used for flow cytometry analysis (see 4.9. Briefly, 2 mL of blocking buffer were applied onto the membrane and incubated for 30 min at room temperature. Next, 2 mL of the supernatant collected from the control and treated cells were incubated with the membrane overnight at 4 °C. Following a series of washes, a biotinylated antibody cocktail was applied onto the membrane and incubated for 2 h at room temperature. Unbound antibodies were removed with a series of washes, and the membrane was placed in HRP-streptavidin and incubated for 2 h at room temperature. Following a third series of washes, chemiluminescence was detected, and the bound proteins were visualized using an X-ray film. Signal intensities were compared using the ImageJ software (MosaicJ, Philippe Thevenaz): relative differences in the expression levels of each analyzed sample were measured and normalized to the intensities of the positive control using the Protein Array Analyzer plugin. The obtained data were analyzed automatically using the Microsoft^®^ Excel-based Analysis Software Tool for Human Cytokine Antibody Array C1000. The results were calculated as a percentage of expression, with positive control set to 100% and negative control set to 0% (relative expression). The threshold was set to 10%. All results equal to or above 10% were considered as real expression.

### 4.11. Statistical Analysis

All statistical analyses were performed using GraphPad Prism version 7 (GraphPad Software Inc., San Diego, CA, USA). The data were compared using the one-way ANOVA test with Dunnett’s multiple comparison. All results are presented as mean ± SEM values.

## Abbreviations

ATMSC	Adipose tissue-derived mesenchymal stem cell
BM-MSC	Bone marrow -derived mesenchymal stem cell
CSC	Cancer stem cells
DMEM	Dulbecco’s modified Eagle’s medium
EGF	Epidermal growth factor
FBS	Fetal bovine serum
FGF	Fibroblast growth factor
FITC	Fluorescein isothiocyanate
GRO	Growth- regulated oncogene, CXCL1 chemokine
HATMSC	Human adipose tissue mesenchymal stem cell line
IL	Interleukin
MCP-1	Macrophage chemoattractant protein-1, CCL2 chemokine
MVs	Microvesicles
MTT	(3-(4,5-Dimetylthiazol-2-yl)-2,5-Diphenyltetrazolium Bromide
PE	Phycoerythrin
RANTES	Regulated on Activation, Normal T Cell Expressed and Secreted, CCL5 chemokine
SSEA4	Stage Specific Embryonic Antigen 4
UC-MSC	Umbilical cord -derived mesenchymal stem cell
VEGF	Vascular endothelial growth factor
